# The impact of global falsified medicines regulation on healthcare stakeholders in the legitimate pharmaceutical supply chain: a systematic review

**DOI:** 10.3389/fmed.2024.1429872

**Published:** 2024-07-18

**Authors:** Ellen Melia, Aislinn English, Bernard D. Naughton

**Affiliations:** ^1^The School of Pharmacy and Pharmaceutical Sciences, Trinity College Dublin, The University of Dublin, Dublin, Ireland; ^2^The Centre for Pharmaceutical Medicine Research, Kings College London, London, United Kingdom

**Keywords:** falsified medicines, counterfeit medicines, regulation, Falsified Medicines Directive, healthcare, pharmacy, impact

## Abstract

**Background:**

Falsified medicines and their international regulation impacts all healthcare sectors and their actors. These regulations aim to strengthen and protect the global pharmaceutical supply chain against falsified medicines. However, an evaluation of the impacts of these regulations on key stakeholders within the legitimate supply chain have not been explored.

**Objective:**

This research aimed to evaluate both the positive and negative impacts of falsified medicines regulation on key stakeholders within the global pharmacy sector including including manufacturers, wholesalers, hospital pharmacies, community pharmacy and patients.

**Design:**

This research consists of a systematic review and thematic analysis concerning falsified medicines regulation and the subsequent impacts of existing global regulations on healthcare. The Preferred Reporting Items for Systematic Reviews and Meta-Analyses (PRISMA) statement and checklist were utilized for reporting in this systematic review.

**Data sources and methods:**

A search of three databases, Embase, ProQuest and PubMed, was undertaken to determine studies applicable to the research question. The Mixed Methods Appraisal Tool (MMAT) was used to assess methodological quality and risk of bias for all included studies.

**Results:**

From the initial 657 studies, a final set of 13 relevant studies were identified. The most frequently reported falsified medicines regulation was the Falsified Medicines Directive (FMD) [*n* = 11]. The impact of falsified medicines regulation in the literature related to four areas: (1) Financial, (2) Social, (3) Organizational, and (4) Pharmacy Practice. These common themes across the included studies frequently relate to challenges and/or concerns associated with falsified medicines regulation implementation as well as both the logistics and practicality of incorporating falsified medicines regulations into daily operations.

**Conclusion:**

Implementation and enforcement of falsified medicines regulation does not yet appear to categorically fulfill the primary aim of the regulations, to strengthen the drug supply chain. However, in recent years, such regulations have challenged the legitimate pharmaceutical supply change actors as they attempt to successfully implement these regulations. Studies mainly detail the negative impacts of regulation during the implementation phase but with the overall benefit pertaining to the prioritization and enhancement of patient care and safety within the healthcare sector.

## Introduction

Falsified medicines are fraudulent medicinal products developed to mimic the origin, constituents, and identity of authorized medicines (1, 2). The prevalence of substandard and falsified (SF) medicines globally is contested in the literature due to different studies calculating prevalence in the presence of different contextual factors. However, best estimates suggest that between 10.5 and 25% of global medicines are SF (3–7) with much fewer examples existing in higher income countries (8–10). Across the world, there are up to 2 billion individuals for whom basic medicinal products are inaccessible (11). Those from low-income countries are notably affected due to the unavailability and unaffordability of life saving medication including antibiotics and antiviral drugs used to treat tuberculosis, malaria, HIV and AIDS (12, 13). Due to this access issue, there is a ‘void’ which falsified medicines fill, resulting in an increased prominence of unsafe, falsified medication entering the supply chain. This demand “void” in the presence of a weak regulatory framework, oversight and enforcement is worrying. Even OECD countries with strong regulatory frameworks experience demand voids which are filled by online medicine purchases. With this in mind regulators need to practice dynamic policy making over time.

It is important to note that falsified medicines are not limited to areas of poor socioeconomic status as global medication shortages and increased popularity of ‘lifestyle’ medications have also contributed to issues in sourcing authorized products across the globe, resulting in medication price escalation (2).

The detection of falsified medicines has improved over the last 10 years due to the introduction of verification or ‘track and trace’ systems that record a products movement within the supply chain, from time of manufacture until reaching the end user. A unique 2-dimensional barcode identifier and anti-tamper device have elevated the level of security within the supply chain, rendering the infiltration of illegitimate medicines more difficult to achieve (14–17). In the EU, most prescription medicines and a portion of over-the-counter medicines are traced through the verification of the unique product packaging code through scanning devices and results in product decommission at the point prior to the medicine reaching the patient, the exact process differs slightly depending on each specific region and regulation (14–16). Through this process, movements associated with each medicine are tracked and recorded on a central database. This allows for full transparency, identification of authenticity and strengthening of the supply chain.

A global effort has been made through falsified medicines regulation to diminish the presence of falsified medicines internationally. Although the current focus of these regulations and technologies are on higher income countries, the implementation of these regulations and associated technologies are also possible in lower income countries once suitable regulatory and technological infrastructure is in place (18). The regulation of falsified medicine aims to preserve patient health and safety, impede the entrance of fraudulent medicines into the supply chain and strengthen the security surrounding the manufacture, delivery, and supply of products (15). One of the earliest regulations concerning falsified medicines emerged in Europe in 2011 in the form of the Falsified Medicines Directive (FMD) and the Drug Supply Chain Security Act (DSCSA) in 2013 in the United States since (19, 20). Laws such as these aid in the detection of fraudulent medicines and limit the risk of substandard drugs reaching patients.

In 2023, the importance of these regulations was seen when falsified semaglutide (Ozempic®) was identified in Germany through the FMD system (21). On scanning, inactive serial numbers were identified which alerted the operator to the possible presence of falsified goods. The origin of the products could be traced back to Austrian and German wholesalers through the EU-wide electronic system (21). Semaglutide is used for weight loss and in the treatment of type 2 diabetes mellitus with global shortages of the drug in recent months due to increased supply demand. The infiltration of this false medication into the supply chain, but more importantly, the detection of this falsified medication signifies the need for regulation.

Falsified medicines risk three key areas, quality, safety, and efficacy that would be reassured with an authorized medicine. The absence of these key areas can cause direct patient harm. The regulations and directives concerning falsified medicines optimize these imperative characteristics and certify medicine authenticity. Falsified medicines and associated regulations impact all stakeholders within the supply chain; manufacturers, wholesalers, hospital, and community pharmacies where the patient is the end recipient. The patient is the main priority for all healthcare professionals and counterfeit (US term) and falsified (EU term) medicines jeopardize patient health and safety. In the United States, the term ‘counterfeit’ is still used in reference to ‘falsified’ or ‘fake’ medicines but within Europe, this term is not used as it refers to intellectual property (IP) infringements rather than medicines designed to imitate that of the authentic pharmaceutical (2, 22).

Both FMD and the DSCSA aim to secure the pharmaceutical supply chain, however, both regulations differ as the DSCSA is a full track and trace process (20) but within the EU, the process slightly differs, involving end-to-end scanning under FMD (19). In Europe, the onus lies upon preventing the entry of falsified medicines into the supply chain, through the use of a unique identifier and an anti-tampering device (19). Whereas within the United States, the DSCSA focuses upon the electronic track and trace of prescription pharmaceuticals as they move through the supply chain with no requirement for pharmaceuticals to bear an anti-tampering device (20).

Falsified medicine research is multifaceted and encompasses policy, governance, public health, and technology researchers amongst others. Published studies have investigated both the positive and negative impacts of falsified medicine regulation on healthcare. The common theme associated with falsified medicine regulation and studies surrounding the topic, is the protection of patient health through strengthening of the global supply chain. However, there are arguments against the implementation of these regulations. For example, the FMD system requires time, workforce and financial resources depending on good internet connectivity, scanning of all packages supplied by staff in a timely fashion as well as dealing with the technical issues that impede workflow and distribution efficiency (23).

This topic is even more relevant due to current discourse regarding the perceived usefulness of the regulations as well as current and recent global factors, including the COVID-19 pandemic, global medication shortages and worldwide medicine price escalation which either directly or indirectly contribute to the prominence of falsified medicines in today’s climate.

The primary aim of this research was to conduct a systematic review and thematic analysis to evaluate the impact of falsified medicines regulation on legitimate stakeholders in the pharmaceutical supply chain. The objective of this study was to investigate the impact of falsified medicines regulation on four principal legitimate stakeholder groups,; manufacturers, wholesalers, hospital, and community pharmacies and to identify the extent of research in this area. The study did not aim to analyze or discuss the impact of these regulations on illegal or illegitimate stakeholder groups. The ultimate goals of this study being to illustrate from the published research, the impact of these regulations on the relevant health sectors, uncover under explored research areas for falsified medicines regulation and to establish future recommendations to enhance the regulation of falsified medicines.

## Methodology

### Overview

To certify that a high-quality systematic review was performed, multiple techniques were utilized to ensure reliability and creditability of both methods and results. The review process was initiated through the search of three databases that included: Embase, ProQuest and PubMed. This was followed by the removal of duplicates and subsequent retrieval of finalized records for screening. Each database search employed a collection of specified search terms relating to the research question, to extract all relevant results. Papers were screened according to predefined eligibility criteria which led to study exclusion or inclusion. Studies included following completion of title and abstract screening, underwent full text appraisal. The entire methodology for this review was performed in accordance with the PRISMA 2020 checklist for systematic reviews (24, 25).

### Search strategy

Guidelines detailed within the Cochrane Handbook for Systematic Reviews of Interventions (26) were followed throughout the process, ensuring compliance with pre-specified eligibility criteria. The PRISMA Checklist for both systematic reviews and the abstract for systematic reviews was followed to certify complete clarity and transparency (see Supplementary Tables 1, 2).

Key search terms were developed based on the research question. Key words and associated search terms were identified through reviewer discussion and the Population, Intervention, Comparison, and Outcome (PICO) framework (27). Particular focus surrounded four main concepts of the research topic and their respective synonyms; (i) falsified, (ii) medicine, (iii) regulation, and (iv) impact, the results of which are contained within Supplementary Table 1. Key terms were searched individually in each database to broaden the search scope. Search filters were utilized to refine and retrieve accurate results. Searching combinations of keywords increased result sensitivity and specificity. Material which contained combinations of key search terms within the title or abstract were incorporated into the review process.

Eligibility criteria for this review included any papers that evaluated the regulation of falsified medicines between January 2010 and February 2024. All included research articles were published in English, had full text publication access and were available to the reviewers. Studies were excluded if published before 2010 and written in any language other than English. Papers with restricted access and any studies comprising of secondary data in the form of commentaries, editorials, letters, notes, reports, or reviews were also excluded. Non-relevant content and duplicated study results were also eliminated from the process.

### Data sources

The search strategy was implemented on three databases: Embase, ProQuest and PubMed. This choice of databases was made in recognition that the research question not only centers around health sciences but also encompasses social science in terms of law and policy, bringing a different perspective to the review and ensuring no relevant studies applicable to the review were overlooked.

Upon finalizing the included research articles from the database searches, a citation search of all references contained within the included studies was undertaken to identify further applicable studies not captured within the initial search. This process was undertaken independently by both reviewers, results compared upon point of completion, with any conflict discussed and resolved between both reviewers to reach a mutual decision regarding inclusion. The suitable studies that were retrieved from this process were included, in addition to the included studies sourced from the initial database searches.

### Study selection and data extraction

All final recognized citations from each database were imported into the selected reference management software, EndNote. Any duplicated studies were identified and excluded.

The PRISMA 2020 Checklist was used as the template to guide the screening process. Covidence® was employed as the systematic review tool to facilitate study selection and data extraction. Both the title and abstract of each identified study were independently screened by two reviewers (EM & AE), assessing the paper for full text inspection.

The finalized title and abstract dataset underwent full text screening to ensure compliance with the pre-established inclusion and exclusion criteria. Full text screening was conducted independently by both reviewers, results compared, and conflicts resolved through discussion. The third reviewer (BN) was consulted to aid in conflict resolution where mutual agreement could not be achieved or where confusion arose regarding eligibility. No reviewer was blind to the journal or author from which the research articles originated.

Texts which did not meet the inclusion criteria were eliminated from the review. The full texts of included studies, deemed relevant and reliable, proceeded to the data extraction stage. Data was evaluated based on risk of bias criteria and study characteristics extracted related to the research question. The following data was extracted and tabulated; year of publication, geographical region/location, study aim and/or design, specified regulation, challenges and impacts of falsified medicines regulations on key stakeholders, as seen in Supplementary Table 2.

### Data Analysis and Risk of Bias

Identified patterns from the study data were subsequently defined and compared under the main objectives of this research paper. In addition to this, a risk of bias assessment was conducted for each paper included in the review to guarantee transparency of all research findings and results. The bias risk for this systematic review was assessed using the mixed methods appraisal tool (MMAT) that allowed for the appraisal of 25 criteria across five different data categories, using the criterion indicators to evaluate the bias and methodological quality of each included study (28). Both reviewers independently conducted the assessment process and generated a total bias score for each study. The results of the risk of bias assessment for both reviewers were compared and discussed. Subsequently, the risk of bias assessment data was tabulated and added to the extracted data form for the review.

## Results

### Study selection and characteristics

**Figure 1:**
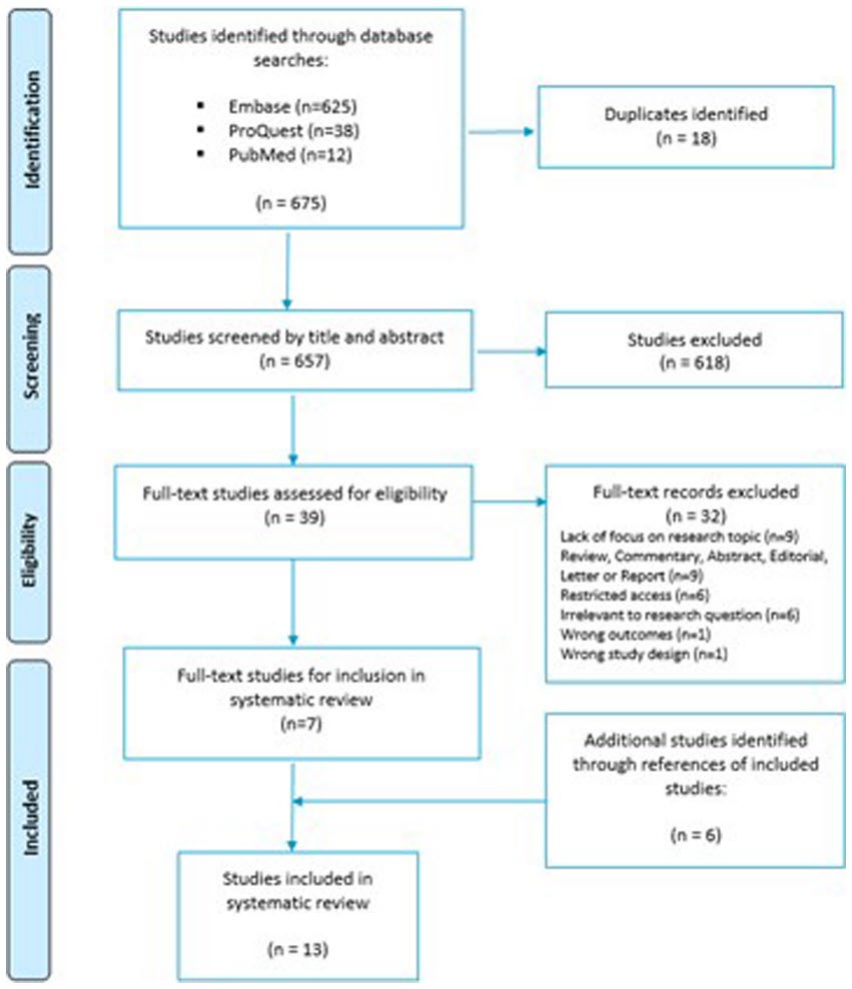
PRISMA flow diagram of the search and review process.

The initial search of the selected databases produced a total yield of 675 articles for screening. The three databases generated the following number of individualized search hits: Embase [*n* = 625], ProQuest [*n* = 38] and PubMed [*n* = 12]. After the removal of duplicate studies, the total number brought forward for title and abstract screening equated to 657 studies. Among the 657 studies, 618 were excluded following the completion of title and abstract screening by both reviewers. Full text analysis was conducted on 39 articles. Of the 39 articles that underwent full text screening, seven articles were chosen for data extraction, risk of bias assessment and thematic analysis after the exclusion criteria were applied. Thirty-two records were excluded based on lack of focus upon research topic (*n* = 9), wrong document type; review, commentary, abstract, editorial, letter or report (*n* = 9), restricted access to the article (*n* = 6), irrelevance to the research question (*n* = 6), wrong study outcomes (*n* = 1) and wrong study design (*n* = 1). A PRISMA flow diagram representing the search and review process is presented in Figure 1.

Following the determination of seven articles for inclusion, a forward citation search was conducted to identify further studies that were not captured as part of the initial searches. Twenty-seven potential studies were identified from the search of bibliography contained within included papers. Screening the bibliographies of included papers is standard practice in a systematic review and ensures that all papers relevant to the research question are included. The additional six studies may not have been picked up in the original study screen for a variety of reasons, e.g. due to being published in journals outside the existing databases. After independent screening by both reviewers, conflict resolution and application of exclusion criteria, six further articles were finalized for inclusion in addition to the seven previous studies. A final total of 13 articles passed quality assessment and were subsequently included in this systematic review.

### Synthesized findings

**Figure 2:**
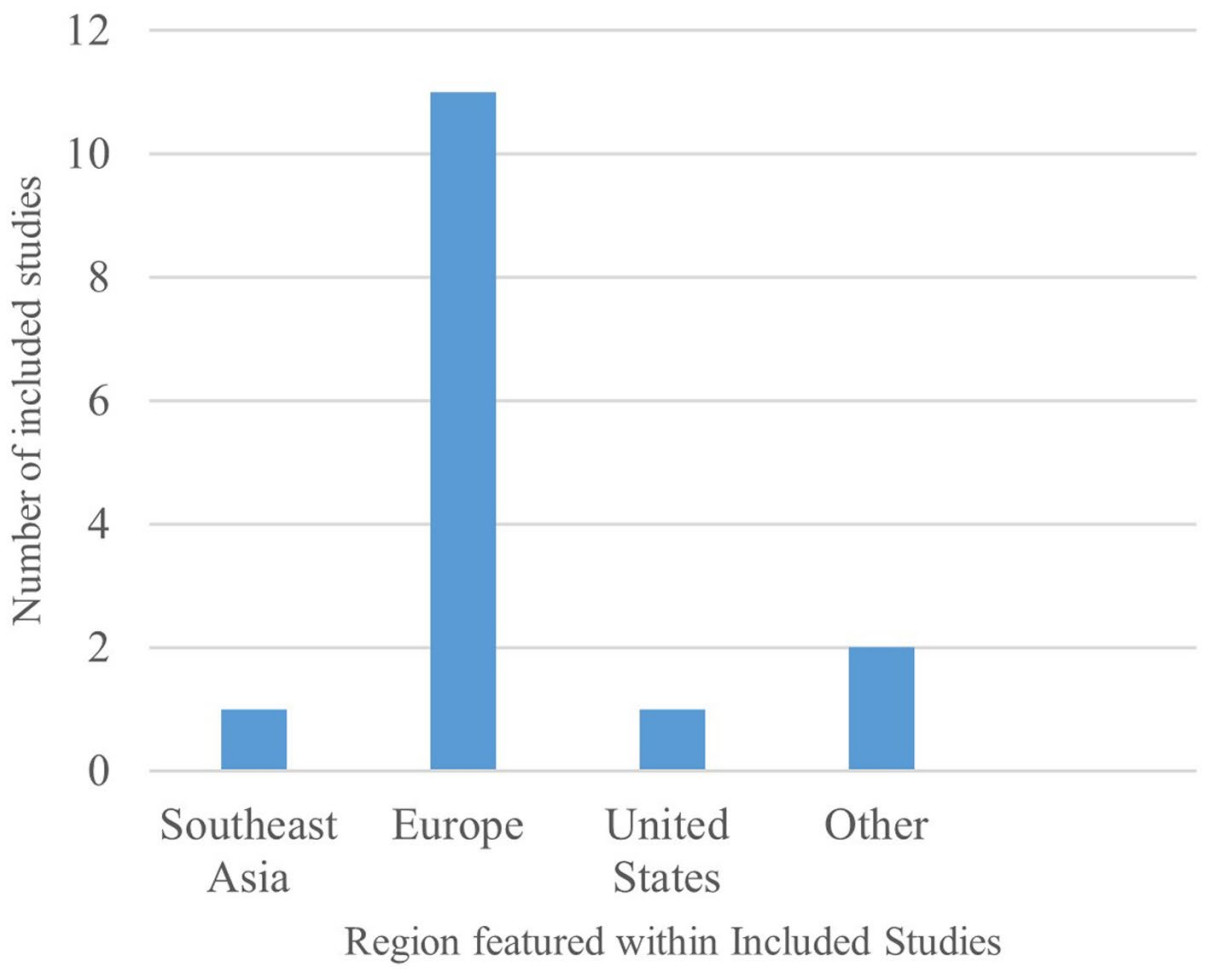
Geographical distribution within included studies.

Across all 13 included studies, identified, most studies explored falsified medicines in countries within Europe [*n* = 11] (16, 29–38), including Austria, Denmark, England, France, Germany, Hungary, Ireland, Poland, and the United Kingdom, as seen in Figure 2. As a result, the majority of research recorded in this review encompasses the FMD, the impact surrounding FMD implementation and continued FMD operation on key pharmacy stakeholders within these countries. However, some featured articles do refer to regulations in Southeast Asia, Brazil, the United States, and Japan but with both references to the DSCSA and the Japanese Encoding of Pharmaceuticals laws featuring within studies also detailing FMD.

Although the research in this area focuses heavily on the impact of the FMD, this data covers a broad range of countries across a varied selection of stakeholders of interest. Such data provides a detailed insight into the true effects of a regulation combatting falsified medicines and allows for comparison across countries in the same region for identification of common trends associated with regulation success and failure.

In Supplementary Table 2, the prominent stakeholders addressed within included studies is hospital pharmacy [*n* = 8] (29–34, 36, 37) and the patient [*n* = 6] (16, 29, 30, 35, 38, 39). Research articles greatly focused upon studies conducted within hospital pharmacy and the impact of FMD on secondary care environments. Subsequently, the patient also featured frequently within studies due to the patient being directly affected by both the positive and negative impacts of FMD introduction into the secondary care environment and FMD impacting all stages in the process prior to the medicine reaching the end user. Less research surfaced surrounding pharmaceutical industry stakeholders and community pharmacies.

**Figure 3:**
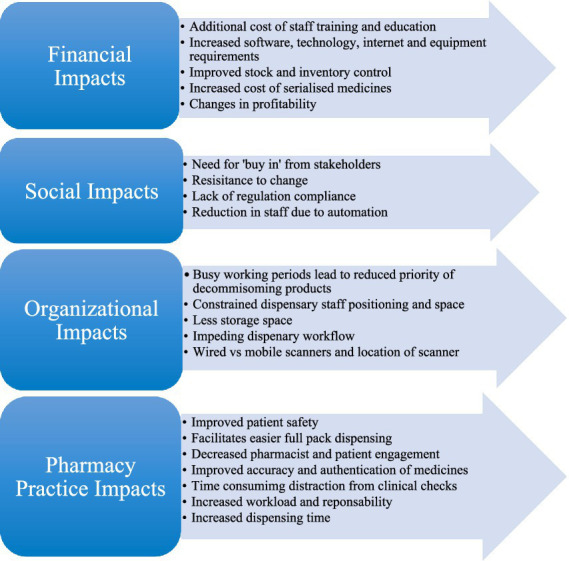
Summary of the main primary and secondary impact themes within included studies.

Investigating the impact of the regulations upon stakeholders unearthed research relating to both the positive and negative effects of FMD and associated laws on all impacted stakeholders. These findings can be divided into four main themes: (1) Financial Impacts, (2) Social Impacts, (3) Organizational Impacts, and (4) Pharmacy Practice Impacts. A summary of all the main impact findings under these themes is denoted within Figure 3.

The detailed results pertaining to the data extracted from all included studies are presented in Supplementary Table 2. The results cover the following elements: article author, year of article publication, study aim or design, challenge(s) associated with regulations, key stakeholder(s), discussed falsified medicines regulation(s) within the study, the impacts associated with the regulation(s) on the stakeholders of interest.

The primary interest in this review was in identifying the impact of falsified medicines regulation, based on the content of included studies, on the stakeholders of interest. In extracting this data, the challenges and barriers faced by stakeholders regarding fraudulent medicines regulation is evidently broad (Supplementary Table 2). On examination of results, the number and type of challenges faced by stakeholders can affect the impacts of the regulation, both positive and/or negative, experienced by stakeholders.

### Assessment of risk of bias

The MMAT was adopted as the risk of bias assessment tool to individually assess the methodological quality of each included article (28). Examining the included studies using the MMAT aimed to minimize research bias from articles that showcase poor collection methods. The MMAT assesses 25 criteria across five different study categories, including: (1) qualitative, (2) randomized controlled, (3) quantitative non-randomized, (4) quantitative descriptive, and (5) mixed methods. For this review, no included studies featured randomized control trials or quantitative non-randomized studies. However, qualitative [*n* = 3] (16, 30, 40), quantitative descriptive [*n* = 8] (29, 31, 33–35, 37–39) and mixed methods [*n* = 2] (32, 36) featured for the finalized dataset.

**Figure 4:**
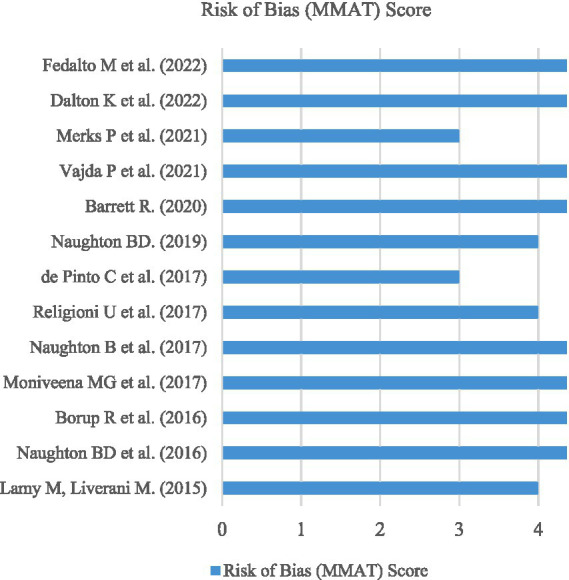
Risk of bias (MMAT) assessment scores for all included studies.

Overall, no studies scored less than 60% (3 out of 5) based on the relevant MMAT study criteria. As a result, all studies included in the review can be described as methodologically sound. A total of eight articles scored full marks according to the relevant MMAT criteria, accounting for over 60% of the total studies contained within this systematic review, as outlined in Figure 4.

## Discussion

This research is one of the first studies to analyze and assess the impact of falsified medicines legislation on specified stakeholders within the pharmaceutical supply chain from the published academic literature. Most stakeholders are aware of the regulations and their aim to protect the supply chain against infiltration of counterfeit or falsified medicines, to improve patient safety and ultimately, to certify medicine authenticity when reaching the end user (41). As seen in Supplementary Table 2, the impacts of the regulations, in particular the FMD, affect the different pharmacy stakeholders in many ways. The same set of regulatory impacts, whether positive or negative, are not present in all settings due to differing stakeholder contexts.

### Ramifications for manufacture and wholesale operations

The EU FMD is described as a “launching pad” to purposely strengthen the supply chain and improve the traceability of pharmaceuticals (42). For manufacturers, tracking systems allow for full transparency and visibility surrounding chain of custody. Serialization and automated electronic methods reduce the likelihood of human input errors and electronic communication enhances the efficiency of reverse logistics procedures (16, 40). Both manufacturers and wholesalers benefit from the streamlined product recall procedure because of the regulations now in place (16, 40). Wholesalers can easily identify theft, product diversion and parallel trade through the improved authentication and detection procedures, reducing feasibility of falsified drug access into the supply chain at an earlier stage (16).

Concerns have arisen regarding the negative implications for manufacture and wholesale distribution. Individual pack scanning requirements as opposed to bulk scanning are significant “practical and costly challenges to the smooth operation of the distribution chain” resulting in slower delivery speed and a longer delivery timeframe for both hospital and community pharmacies and subsequently, the patient (43).

### Consequences for the community pharmacy

Within community pharmacies the main overarching concerns relate to time, disruption, and lack of ‘buy in’ by staff. According to a survey conducted by Dalton et al. (38), participants working within community pharmacy view FMD as a “significant disruption” to workflow in addition to technical issues that “add variation and unpredictability.” A community pharmacy is a high paced, fast turnaround environment and from the data obtained within included studies, the introduction of an additional step in the medication supply process is considered a burden. During busy work periods the requirement to decommission products becomes less of a priority due to the need to gather, dispense and check medication in a timely and safe manner. In addition to this, the scanner location within the dispensary can constrain staff positioning and space, making the scanning of every medication more difficult.

In community pharmacy the patient/pharmacist relationship is crucial, the impact of the FMD on daily pharmacy practice was reported by Dalton et al. (38), highlighting pharmacist’s concerns surrounding reduced time for patient interaction, distraction from clinical checks leading to potential near misses, an increased prescription turnaround time for patients and subsequently, a larger workload for the pharmacist. The FMD process was described by one pharmacist as a “huge amount of work for no real benefit” (38). Additionally, studies reported that there was a belief that manufacturers should be responsible for verification steps and not community pharmacies.

In terms of the positive effects, most studies included in the review related to community pharmacy, acknowledge the improved patient safety associated with the use of the FMD system. In the study by Barrett (35), an overwhelming majority of respondents, accumulating to 77.5%, believed that FMD might improve patient safety. In addition to this, it was highlighted that FMD scanning makes full pack dispensing easier due to the tamper proof seal which emphasizes the enhanced patient safety nature of regulation implementation. Other beneficial opportunities proposed were the potential use of system technology to be utilized for batch recalls, expiry date checking and inventory control.

### Implications for hospital pharmacy

The impacts of falsified medicines regulation within secondary care settings featured prominently as the focus for research within included studies. All studies applicable to the hospital setting addressed the FMD regulation.

Interestingly, surrounding FMD implementation in hospitals, the 10-day decommissioning rule under Article 13, was addressed in multiple studies as a limitation for hospital operation. The regulation refers to the period of 10 days from the point of decommission on a premises where if these 10 days pass, the medicine can be returned to the pharmacy from the wards but can only be used in the physical institution where it was originally decommissioned (32, 37). With this stipulation in the regulation, this requires all parties to be aware of the date of decommission to ensure a product is eligible for transfer to a different institution if required within this time frame. Emergency supplies are also limited as a result.

Similarly to community pharmacy, the FMD was reported to result in high IT, infrastructure and training investment costs and increase the overall workload for both pharmacists and pharmacy technicians. However, the benefits of FMD within hospitals are also important to highlight as the improved authentication and detection rates aid to tighten the supply chain, reducing the risk of falsified medicines circulating within a hospital. The hospital setting is one that uses more single dose units that lack the FMD enhanced safety features, as opposed to secondary product packaging that contains the unique product identifier and tamper proof seal.

In a review by Naughton et al. (44), the criticality of FMD implementation and compliance in secondary care was emphasized for patient safety. It also recognized that return of unused medicines on the wards back into stock does complicate the secondary care drug distribution cycle and thus, complicates the effective implementation of the FMD. However, in the first instance, this practice in combination with multiple drug entry, does promote the entry of falsified medicines into the supply chain (44). Therefore, implementing the FMD should reduce the likelihood of illegitimate medicines lying undetected. In addition to this, it is also highlighted that “whether it is a pharmacist in a dispensary or a nurse on ward level each member of staff must physically pick up each medicine container and check that they have selected the correct product” (44). An extra step, taking 250–300 milliseconds to decommission the product, would be a minor yet important addition to current practice. The anti-tampering device is also acknowledged as providing “improved efficiency to current practice” (44).

In terms of impeding workflow, the data contained within the included articles vary. According to Naughton et al. (31) secondary care study, “there were no concerns regarding the speed and usability of authentication technology” and “there was limited impact on the daily activity of the staff.” Whereas a similar hospital study in Poland in the same year did indicate the FMD as impeding workflow (32). In addition to this, a study was conducted in Austria which did highlight the impact upon dispensing operations, but that significant structure and preparation are required to facilitate proper handling of the decommissioning process (33). This highlights that with thorough planning, the decommissioning process does not have to negatively impact workflow.

### Exploring the patient impact

The overriding benefit of falsified medicines regulation addressed within all included studies relates to the improvement of patient safety. The introduction of falsified medicines legislation by authorities, works to minimize the possibility of any fraudulent pharmaceuticals reaching or being consumed by the end user. These regulations aim to protect the patient which is a global priority for all healthcare professionals and the acceptance of these challenges that stem from the implementation of change for the better, to certify patient safety, is evident from the studies. The long-term advantages of regulatory implementation involve further protection of the patient through a facilitated reduction in medication errors related to the ability of systems to alert the pharmacist to expired stock, product recalls or as seen with Ozempic^®^ in Germany, alerting the pharmacist to potential falsification (16, 38).

### Primary themes across all stakeholders

When analyzing the results across all 13 included studies, the primary themes highlighted in Figure 3 recurrently featured, these include: (1) Financial, (2) Social, (3) Organizational, and (4) Pharmacy Practice Impacts. From a financial perspective, studies indicate that implementation of falsified medicines regulations can be costly. Expenditure accumulates when training, education and infrastructure costs are considered, for some companies the cost of hiring experts in regulatory requirements is also a factor to contemplate (30). In contrast to this, regarding social impacts, studies did highlight that increased automation can lead to a reduction in staff, potentially reducing financial burden and leading to less human input errors and increased accuracy (16, 33). Additionally, increased automation can benefit pharmacy practice by improving inventory control, identifying expired stock, enhancing medication recall effectiveness, and facilitating easier full pack dispensing in community and hospital settings which contributes to improved patient safety (16, 29, 38). This approach has also been recommended at the Member State and EU level, whereby the FMD data networks would be able to help manage stocks by identifying potential products at risk of shortage and where existing supplies are available. With benefits also come disadvantages, serialization does hinder organization within a dispensary. Reduced storage capacity, location of scanners and the introduction of a decommissioning step all impact adjacent processes and disrupt workflow (34–38). Common themes across all included studies emphasize that implementation of falsified medicines regulation is not a seamless process and difficulties are encountered, however, many advantages are also evident.

### Evidence: reviews vs. research

A primary challenge faced with this study involved the lack of original and published research surrounding falsified medicines regulation. This study identified that there was an abundance of reviews and opinions that exist around the topic, making this a contentious area for research, however original research was limited. As seen within the PRISMA flow diagram in Figure 1, 28% (9 out of 32) of excluded studies during the full text screening process were removed due to the study being a review, commentary, abstract, editorial, letter, note or report, even though the content of the publications were relevant to the research question. It is evident that there is a shortage of substantive, primary evidence focusing specifically upon the impact of falsified medicine regulations. The research that does exist in this area is helpful to examine the efficacy of falsified medicine regulations and provides an insight into the reality of implementing falsified medicine regulations, including what has been successful and any shortcomings that exist between written legislation and the practicalities of stakeholders enforcing these regulations.

### Limitations

This systematic review has multiple limitations. Firstly, there were boundaries put on the study. Article language was one limitation as only articles that were written and available in the English language were included within the review. This systematic review was restricted to a timeframe of the previous 14 years, 2010–2024, which was included within the eligibility criteria for the review, limiting the extent of the research captured. Finally, regulatory research is uncommon and the substandard and falsified medicine regulations are relatively new making research in this area scarce.

### Policy and research recommendations

Overall, government policies adopted by each nation represent positive steps toward safer pharmaceutical supply chains, however, they are still in their infancy. These regulations are at a crucial stage of development, and we recommend that policy makers be mindful of the positive and negative aspects of each regulation. We would urge policy makers to be open to adjusting their own policy based on the learnings from other countries with subtly differing regulations. A responsible innovation approach, which includes anticipatory governance, inclusion of suitable stakeholder groups, reflection on stakeholder feedback, responsivity to stakeholder experiences, and appropriate regulatory impact assessment could be a suitable way to guide the further development of these important regulations (45, 46).

Future research in this area could help to develop the necessary evidence to support impact assessments and regulatory development. Such research could focus more upon conducting primary research relating to falsified medicines regulation, and its impact. More global and advanced primary data would allow researchers to produce a systematic review reflective and applicable to the global healthcare community. In addition to this, considering the prevalence of reviews and opinion pieces that exist, conducting a review of these reviews could be an interesting opportunity to explore the debate that exists between the need for and against the implementation of falsified medicine regulations in their current form, which would help policy makers understand stakeholder narratives more clearly. The lack of research data encountered in the area of falsified medicines regulation is one which is a more wide-spread issue linked to regulatory research more broadly. More research in this field of study would certainly be useful but it cannot be disregarded that empirical regulatory research is not a regular occurrence (47), and more should be done to fund and encourage regulatory research to assess stakeholder impact.

Regarding advancements directly relating to the current regulations, authorities can seek to expand the role of falsified medicines regulations and systems within healthcare. Taking advantage of the opportunity presented by the advanced ‘track and trace’ technology could prevent sudden and extreme worldwide medication shortages, such as the global shortage of Ozempic® experienced in the past year, through the utilization of medication decommissioning data to extrapolate possible incoming drug shortages due to increased supply uptake and/or manufacturing issues (48). In addition to this, benefiting from the already present barcode technology, patient mobile reporting of adverse drug reactions (ADRs) through scanning of a unique barcode could also be considered.

The unique identifier on packaging can also be utilized as an additional opportunity to protect patients as encouraging patients to look out for this barcode on medicine packaging could aid in identifying falsified products, especially in the case of patients purchasing from online sources themselves (44). Even though alerting and educating patients to the presence and meaning of falsified medicines and the authentication barcode will take considerable time and dedication, the potential for this to prove effective does exist and could be incorporated as a part of electronic patient information leaflets (44). For this to be successful in the future, the issues currently faced by other stakeholders within the supply chain, other than the patient, would need to be addressed to ensure a strong foundation to explore the broader scope of the FMD, in which it can be further used to enhance patient safety and enlighten patients to the existence and prominence of falsified medicines.

Additionally, expanding the detail and scope of serialization for the single dose unit 2D Data Matrix to appear not only on secondary packaging but also to compulsory feature on all primary medicine packaging would further enhance traceability and certify authenticity of all medicines within pharmacy settings where all products are handled as single dose units such as the inpatient hospital environment.

## Conclusion

Falsified and substandard medicines remain a problem in the world today. However, the introduction of falsified medicines regulations has contributed to a strengthened pharmaceutical supply chain with reduced likelihood of fraudulent medicines entering or laying undetected within the supply chain itself. The main challenges faced by key stakeholders have arisen during the implementation phase of these regulations. Primary concerns relate to the financial investment and changes imposed to former pharmacy practices and environments. Although studies report numerous challenges faced by stakeholders upon the introduction of falsified medicines legislation, it cannot be denied that such regulation works to improve accuracy, safety and quality associated with all medicines that reach the patient.

This research highlights that irrespective of the obstacles faced while navigating the real-life application of falsified medicines legislation, all stakeholders within the pharmacy sector recognize the importance of prioritizing patient care and safety above all else. However, it cannot be overlooked that there are both positives and negatives experienced by all stakeholders. Four primary themes are evident across results: (1) Financial, (2) Social, (3) Organizational, and (4) Pharmacy Practice Impacts. Increased expenditure arising from technology, equipment and training requirements was a common issue among stakeholders with acceptance of regulation implementation in addition to hindered workflow and limited capacity also causing issues among stakeholders. For patient facing roles in community and hospital pharmacy, the impact on daily pharmacy practice is highlighted. Implemented regulations improve patient safety but at a potential cost of less patient engagement and increased prescription turnaround time, ultimately placing more pressure and workload on pharmacists.

This study encourages government authorities, academic researchers, industry, and healthcare stakeholders to recognize the importance and relevance of falsified medicines regulation in optimizing patient health and in investing both time and resources into primary policy and governance research of this area within healthcare.

## Data availability statement

The original contributions presented in the study are included in the Supplementary material, further inquiries can be directed to the corresponding author.

## Author contributions

EM: Methodology, Writing – review & editing, Data curation, Formal analysis, Investigation, Project administration, Software, Writing – original draft. AE: Data curation, Formal analysis, Investigation, Methodology, Project administration, Software, Writing – original draft, Writing – review & editing. BN: Methodology, Writing – review & editing, Conceptualization, Supervision, Validation.
