# Supplementary material for: The impact of global falsified medicines regulation on healthcare stakeholders in the legitimate pharmaceutical supply chain: a systematic review
Source: Front Med (Lausanne). 2024 Jul 18;11:1429872. doi: 10.3389/fmed.2024.1429872 (PMC11291459; doi:10.3389/fmed.2024.1429872)
Supplement: Supplementary file 1 [file Table_1.docx]

| **No.** | **Search term(s)** | **Number of publications identified** | | |
| --- | --- | --- | --- | --- |
|  |  | **Embase** | **ProQuest** | **PubMed** |
| 1 | False | 70,640 | 3,179 | 3,918 |
| 2 | Falsified | 808 | 64 | 20 |
| 3 | Fraudulent | 779 | 440 | 18 |
| 4 | Fake | 1,739 | 995 | 86 |
| 5 | Counterfeit | 1,090 | 159 | 22 |
| 6 | Substandard | 1,660 | 181 | 89 |
| 7 | Tampered | 202 | 25 | 6 |
| 8 | **1 or 2 or 3 or 4 or 5 or 6 or 7** | **76,152** | **4,811** | **4,118** |
| 9 | Medicine(s) | 345,437 | 10,779 | 25,720 |
| 10 | Drug(s) | 931,312 | 13,420 | 74,503 |
| 11 | Treatment(s) | 2,631,995 | 42,680 | 298,745 |
| 12 | Pharmaceutical(s) | 107,166 | 1,850 | 4,755 |
| 13 | **9 or 10 or 11 or 12** | **3,446,900** | **61,915** | **341,878** |
| 14 | Regulation(s) | 488,821 | 17,990 | 10,713 |
| 15 | Law(s) | 66,803 | 36,139 | 831 |
| 16 | Directive(s) | 9,240 | 1,966 | 547 |
| 17 | Act(s) | 3,358,128 | 143,823 | 5,708 |
| 18 | **14 or 15 or 16 or 17** | **3,646,084** | **181,382** | **17,389** |
| 19 | Challenge(s) | 511,153 | 75,871 | 23,925 |
| 20 | Opportunities | 101,185 | 47,957 | 4,947 |
| 21 | Barrier(s) | 240,502 | 20,240 | 14,220 |
| 22 | Facilitators | 20,469 | 4,167 | 2,962 |
| 23 | Impact(s) | 1,089,065 | 103,799 | 93,852 |
| 24 | **19 or 20 or 21 or 22 or 23** | **1,797,715** | **214,071** | **127,045** |
| 25 | **8 and 13 and 18 and 24** | **625** | **38** | **12** |

***Table 1*.** Literature search strategy, search terms results from the three databases (Embase, ProQuest, PubMed)
