# Supplementary material for: The impact of global falsified medicines regulation on healthcare stakeholders in the legitimate pharmaceutical supply chain: a systematic review
Source: Front Med (Lausanne). 2024 Jul 18;11:1429872. doi: 10.3389/fmed.2024.1429872 (PMC11291459; doi:10.3389/fmed.2024.1429872)
Supplement: Supplementary file 2 [file Table_2.docx]

| **Table 2,** Data extraction of results from research articles included in the systematic review (n=13) | | | | | | | | | |
| --- | --- | --- | --- | --- | --- | --- | --- | --- | --- |
| **S. No.** | **Author** | **Year** | **Country/Region** | **Study Aim/Design** | **Challenge(s)** | **Stakeholder(s)** | **Regulation(s)** | **Impact(s) of Regulation** | **Risk of Bias**  **(MMAT)** |
|  | Lamy M, Liverani, M | 2015 | South East Asia-  Mekong;  Myanmar, Thailand,  Lao PDR, Cambodia, Vietnam | Highlighting the strengths and weaknesses of past national intervention responses in combatting falsified medicines.    Examination of institutional frameworks that exist to support increased capacity with a focus on Southeast Asian Nations | Sale of counterfeit medicines in remote, rural communities    Reduced custom controls and increased cross-border exchanges promotes infiltration of falsified medicines.    Lack of general operational and enforcement capacity to monitor supply chains due to inadequate human resources, equipment and infrastructure  Transnational nature of falsified medicines  Funding shortages and unsustainable financial agreements  Trade expansion of pharmaceuticals  Outdated laws and complex pharmaceutical market | Manufacturer     Patient | National Drug Law (1992, Myanmar),    Law on Pharmacy (2005, Vietnam),  Drug Act (2003, Thailand),  Law on the Management of Pharmaceuticals (1996, Cambodia),  Law on Drugs and Medical Products (2000, Lao PDR) | Deterrent against falsified medicines  Improved case tracking and quality control  Movement towards low-cost and easy-to-use quality control detection  Aids to strengthen legal frameworks, operational scope, and governance mechanisms | ◼◼◼◼🞎 |
|  | Naughton BD, Roberts L, Dopson S, Chapman S, Brindley D. | 2016 | United Kingdom | Identification of the authentication and detection rates of serialised medicines to assess the effectiveness of medicines authentication technology in NHS secondary care | Difficulties with implementation of FMD-legislated serialisation and authentication technologies in secondary care of some countries    Non-compliance issue due to increased number of guidelines and protocols required to be adhered to by NHS staff.  Detected counterfeit, recalled and/or expired medicines not always quarantined by staff  Misinterpretation of alerts due to similar content and colour | Hospital pharmacy  Patient | Falsified Medicines Directive (FMD) | Improved authentication and detection rates    Improved patient safety    Impact on policy and key decision makers- need for effective education and training strategies to achieve successful implementation | ◼◼◼◼◼ |
|  | Borup R,  Kaae S,  Minssen T, Traulsen J | 2016 | Denmark | Reviewing the distribution of medicines in Denmark through comparison of legislation before and after Denmark joining the EU  Assessing the impact of EU harmonisation  Evaluating if drastic increases in mandated Falsified Medicines Directive requirements correlate to a new pharmaceutical supply chain governing approach | Highly complex legislation now covers the entire supply chain    Little room to allow for interpretation of FMD legislation to fit local settings      Equal compliance with FMD becoming priority as opposed to protection of public health | Manufacturer  Wholesaler  Community pharmacy  Hospital pharmacy  Patient | Falsified Medicines Directive (FMD) | New approach to manage the pharmaceutical supply chain- more focus surrounding documentation and increased requirements specificity    Different requirements for individual supply chain actors    Complexity of regulation demands compliance- companies hiring experts in regulatory requirement; additional financial costs    Clear checklist for regulation compliance | ◼◼◼◼◼ |
|  | Moniveena MG, Pramod Kumar TM. | 2017 | United States  European Union  France  Japan  India | Inspection of the track and trace regulations in pharmaceutical industry  Examining the impact of track and trace regulations to protect drug distribution chains in status regulated countries and India  Examining the impact of track and trace regulations on the reverse logistics of medicines | Establishing the identity of individual medicinal products  Some medicinal products may be classed as prescription pharmaceuticals in one Member state but not in another | Wholesaler    Manufacturer    Patient | US Federal: Drug Supply Chain Security Act (DSCSA), ‘Track and Trace Act’    California: Epedigree Law Nov.27 2013    European Union: Falsified Medicines Directive (FMD)    France: French CIP13 Coding Legislation   Japan:  Encoding of Pharmaceuticals with Japan Article Number | Serialisation: electronic methods reduces and/or eliminates human input error  Improved accuracy and time efficiency  Reduced medication errors  Improved inventory control  Increased product recall effectiveness  Detection of theft, product diversion and parallel trade  Tracking systems provide full visibility to chain of custody  Electronic communication helps streamline reverse logistics procedure and reduce cycle time | ◼◼◼◼◼ |
|  | Naughton B, Roberts L, Dopson S, Brindley D, Chapman S. | 2017 | United Kingdom | Determination of expert opinion and possible improvements in the hospital setting for Falsified Medicines Directive (FMD) authentication technology | Lack of training and education, contributing to inadequate authentication and detection rates   Unclear differentiation between warning messages leading to misinterpretation of alerts. | Hospital pharmacy | Falsified Medicines Directive (FMD) | Limited impact on daily activity of staff  ‘Not disruptive’ to daily activity | ◼◼◼◼◼ |
|  | Religioni U, Swieczkowski D, Gawronska A, Kowalczuk A, Drozd M., et al | 2017 | Poland | Assess the value of external audits in the context of FMD implementation regulations in the secondary care setting | Ensuring products are not authenticated too early in the supply chain  Dispensing drugs in bulk- better to select products from manufacturers that produce aggregated codes as if not, will increase product authentication time  10-day decommissioning rule  Split packs- authentication needed prior to opening package and not transferred to other institution | Hospital Pharmacy | Falsified Medicines Directive (FMD)  Delegated Act (DA) | FMD impeding workflow in the hospital pharmacy  New responsibilities on pharmacists and pharmacy technicians  Unused drugs must be returned to hospital pharmacy from the wards within 10 days of product decommission in order to be eligible for transfer to a different institution  Extemporaneously compounded medicines, intravenous and parenteral nutrition products need to be authenticated during assembly before final product is prepared | ◼◼◼◼🞎 |
|  | de Pinto C, Koshkouei M, Jeske M, Zeiler M, Brindley D. | 2017 | Austria | Appraise the impact of implementation of FMD legal requirements in an Austrian hospital, using a medicines authentication system (MAS). | Implementation of MAS could result in significant disruptions and large upheaval for secondary care pharmacy staff when not handled properly  Connectivity issues | Hospital Pharmacy | Falsified Medicines Directive (FMD) | Impact on dispensing operations- to handle the decommissioning, significantly more structure and preparation is required.    Reduction in staff required in FMD environment due to automation.    Increase in operational dispensing time in a FMD environment  Increased workload - single unique identifier for each product must be decommissioned separately  Internet connectivity issues and national repository location can affect response time  Increased expenditure | ◼◼◼🞎🞎 |
|  | Naughton BD | 2019 | United Kingdom | Investigation of serialised medicines introduction into an operational hospital dispensary and the assessment of technical effectiveness of digital medicine authentication (MA) technology under European Union Falsified Medicines Directive (EU FMD) conditions | Disruptions to healthcare providers if FMD legislation is poorly implemented- key technical parameters including offline issues, incorrect quarantine, and average response times    Low levels of awareness and understanding of FMD regulation among healthcare providers  Inadequately designed technology alerts | Hospital pharmacy | Falsified Medicines Directive (FMD) | FMD offline issues may cause significant delays in medicine supply, a backlog of dispensing and cause confusion at point of decommissioning the product- legal and practical impact  MA is an additional step that impacts adjacent processes | ◼◼◼◼🞎 |
|  | Barrett R | 2020 | England | Assesses the readiness of community pharmacies for the introduction of the Falsified Medicines Directive (FMD) | Lack of resources, knowledge, competency, training, and confidence in pharmacy personnel  Lack of compliance with FMD implementation | Community pharmacy  Patient | Falsified Medicines Directive (FMD) | Negative impact on workload  Disruption to normal business flow    Adds to administrative burden  Changes in profitability  Improves patient safety | ◼◼◼◼◼ |
|  | Vajda P, Richter K, Bodrogi Z, et al. | 2021 | Hungary | Evaluation of the current practices, cost, and workload implications of FMD implementation in Hungarian hospitals within the stabilisation period | Implementation and maintenance costs of verification and decommissioning    Staff resource issues  Pharmacists struggle with different codes and false alarms | Hospital pharmacy | Falsified Medicines Directive (FMD) | Tightens the legal drug supply chain  High implementation and operational costs- IT and infrastructure investment  Additional human resources required in hospitals    Impedes workflow  Price increase in serialised medications  Drug supply issues under FMD  Increased storage capacity requirements- differences in product packaging size under FMD  Increased pharmacist and pharmacy technician workload  Increase in FMD related premises- financial burden | ◼◼◼◼◼ |
|  | Merks P, Religioni U,  Pinto De Castro N,  Drozd M, Mack C,  Świeczkowski D,  Zerhau M, Gawrońska A, Kowalczuk A,  Jaguszewski M, Brindley D, Hug M. | 2021 | Germany | Determination of additional operating time for internal workflow to comply with FMD and establishment of suitable areas to conduct efficient medicine packaging scanning | Establishing the optimal operational procedure for decommissioning  Determining when scanning requires the least operational time  Product packaging- inability to scan through plastic covering, barcode printed at different points of package depending on manufacturer leading to inaccessibility and delays  Type of scanning equipment- wired vs mobile scanner  Article 13, 10-day decommissioning rule | Hospital pharmacy | Falsified Medicines Directive  (FMD)    Delegated Regulation (DR) | Additional workload on hospital pharmacy staff  Point of decommission and its impact on workflow (optimal point is authentication of products before sending to the ward)  Scanning and location of barcode increases operational time on daily basis  10-day decommissioning rule limits emergency loans and supply to other hospitals | ◼◼◼🞎🞎 |
|  | Dalton, K, Connery C, Murphy KD, O’Neill D | 2022 | Ireland | Examination of community pharmacists’ views on how FMD has affected their practice. | Lack of regulation compliance- belief that manufacturers should be responsible for the verification steps    Hardware and software issues- add variation and unpredictability  Busy work periods- reduced priority of decommissioning products  Scanner location- constrained dispensary staff positioning and space  Increased product packaging size due to 2D barcode - storage issues, less information on packaging when pharmacist checking medicine | Community Pharmacy    Patient | Falsified Medicines Directive (FMD) | Significant disruption to dispensing process    Loss in staff productivity    Additional implementation costs- training, software, and equipment    Time consuming distraction to clinical checks, leading to increased risk of dispensing and/or near miss errors    Reduced patient interaction time    Increased prescription turnaround time for patients    Increased workload  Increased technical support requirement  Easier full pack dispensing  Increased patient safety- advantage to track medicines at batch recalls and expiry date check  Benefits stock control process | ◼◼◼◼◼ |
|  | Fedalto M, Stumpf Tonin F, Borba H, Lins Ferreira V, Correr C, Fernandez-Llimos F, Pontarolo R | 2022 | Brazil | Investigating the practice of pharmacists relating to pharmacovigilance activities  Identifying difficulties and possible stimuli to improve these activities in pharmacies and drugstores | Low numbers of pharmacists reporting suspected substandard medicines to Notivisa  Lack of motivation to notify the system  Pharmacists unsure of the cause of the problem  Pharmacists unconvinced by the confidential handling of reported information  Difficult for pharmacists to admit that patients have been harmed  Insufficient clinical and technological medicines knowledge  Pharmacists fear being legally responsible for the issues  Varied knowledge regarding Notivisa- association between education level and Notivisa knowledge, better knowledge associated with a master’s degree to post-doctorate level, specialization, and professional residency  Complexity of making a Notivisa notification  Lack of access to the system at work  Not knowing the email address to send a report to  Unaware of how to notify the system | Community pharmacy  Patient | National Health Surveillance Notification System (Notivisa) | Pharmacists agree with pharmacovigilance importance in protecting public and patient health  Reporting services recognized as part of pharmacist duties and pharmaceutical care  Making a notification is a time-consuming task– impedes workflow and increases turnaround time  Need for additional material, education, courses, and training surrounding the system- requires both time and funds | ◼◼◼◼◼ |
